# Tea Bud Detection Model in a Real Picking Environment Based on an Improved YOLOv5

**DOI:** 10.3390/biomimetics9110692

**Published:** 2024-11-13

**Authors:** Hongfei Li, Min Kong, Yun Shi

**Affiliations:** 1 School of Electrical Engineering, Anhui Polytechnic University, Wuhu 241000, China; 18096733975@163.com; 2 School of Electrical and Photoelectronic Engineering, West Anhui University, Lu’an 237012, China

**Keywords:** tea bud detection, YOLOv5, deep learning, bidirectional feature pyramid

## Abstract

The detection of tea bud targets is the foundation of automated picking of premium tea. This article proposes a high-performance tea bud detection model to address issues such as complex environments, small target tea buds, and blurry device focus in tea bud detection. During the spring tea-picking stage, we collect tea bud images from mountainous tea gardens and annotate them. YOLOv5 tea is an improvement based on YOLOv5, which uses the efficient Simplified Spatial Pyramid Pooling Fast (SimSPPF) in the backbone for easy deployment on tea bud-picking equipment. The neck network adopts the Bidirectional Feature Pyramid Network (BiFPN) structure. It fully integrates deep and shallow feature information, achieving the effect of fusing features at different scales and improving the detection accuracy of focused fuzzy tea buds. It replaces the independent CBS convolution module in traditional neck networks with Omni-Dimensional Dynamic Convolution (ODConv), processing different weights from spatial size, input channel, output channel, and convolution kernel to improve the detection of small targets and occluded tea buds. The experimental results show that the improved model has improved *precision*, *recall*, and mean average *precision* by 4.4%, 2.3%, and 3.2%, respectively, compared to the initial model, and the inference speed of the model has also been improved. This study has theoretical and practical significance for tea bud harvesting in complex environments.

## 1. Introduction

China is the world’s largest producer and consumer of tea, with a wide variety of tea varieties, and holds a high position among the world’s top tea suppliers [1,2]. With the improvement in people’s quality of life, tea, which contains rich active compounds, has gradually become a popular beverage [3]. According to statistics, the tea plantation area in China exceeds 3.27 million hectares, with an annual output of over 3.1 million tons, accounting for more than 40% of the world’s tea production and ranking first in the world [4]. But currently, the picking of tea buds is facing a challenge. Currently, tea bud picking is mainly performed manually, resulting in low picking efficiency and high costs. Traditional tea-picking machines are unable to accurately locate tea buds and can cause tree branches and leaves to break, which not only affects the subsequent growth of tea trees but also reduces the overall quality of the picked tea buds [5,6]. Therefore, accurate target detection of tender buds is the foundation of automated picking of premium tea. However, the variety of tea leaves, the similarity between tea buds and tea characteristics, and the small target size make it easy to be obscured, making tea bud localization and harvesting more challenging. If applied to other similar agricultural scenarios, tea bud target detection becomes more practically valuable.

Traditional image processing research mainly distinguishes tea buds from the background based on features such as color differences, texture, and shape. In the study by Zhang et al. [7], adaptive thresholding is performed using the blue component of the image, which is combined with the green component of the image to form a new grayscale image. The contrast between tender buds and leaves is improved through linear transformation, and a watershed segmentation algorithm is used to locate and identify tea buds. In the study by Huang et al. [8], a partial differential equation model is used to filter out noise in the image, and the watershed algorithm and OTSU algorithm are used to segment the preprocessed image. In the study by Shao et al. [9], histogram equalization was performed using tea bud images. Then, k-means clustering analysis was performed on the saturation components under the HIS color model to obtain the best value of k. In the study by Karunasena et al. [10], gradient histograms of positive and negative samples were trained on tender shoots of different lengths using a Cascade classifier. In the study by Li et al. [11], RGB images of tea buds are extracted, and the LBP/C algorithm is used to extract the texture and shape of tender buds, combined with a support vector machine tea bud localization recognition algorithm to complete model training. However, traditional machine learning algorithms are subject to excessive human intervention in feature extraction, which makes it easy to overlook some features.

In recent years, deep learning technology has been continuously developed and iterated, and through semantic segmentation and object detection algorithms, it can detect and recognize crops by applying these methods to related agricultural fields. In the study by Chen et al. [12], the Faster R-CNN is used to detect the one-bud and two-leaf regions in the image, and then the trained fully convolutional network is used to identify the one-bud and two-leaf regions and locate the picking point. In the study by Yan et al. [13], a tea bud picking point localization method based on Mask R-CNN is proposed, which segments and locates tea buds by training a model to predict picking point positions. In the study by Xu et al. [14], YOLOv3, which has the advantage of fast detection, and DeniseNet201, which has high-*precision* classification ability, are used to achieve precise localization of tea buds. In the study by Li et al. [15], an attention module is embedded in the YOLOv4 network, and the penalty index is redefined using the SIoU loss function. This improves detection accuracy while reducing model size and lowering the deployment cost and difficulty of the robot vision module. In the study by Shuai et al. [16], in the YOLOv5 network, Bottleneck Transformers are used as residual modules, combined with CARAFE and attention mechanisms, to create long-range dependencies on tea bud feature images. In the study by Xie et al. [17], the Tea-YOLOv8s model combines deformable convolutions, attention mechanisms, and improved Spatial Pyramid Pooling, thereby resulting in improved detection *precision*. At present, most methods for tea bud target detection are based on original models, and dataset creation is mostly based on experimental fields, which results in poor applicability to actual tea planting environments. Deep learning-based tea bud object detection still has disadvantages such as low detection efficiency, large model size, poor accuracy, and difficulty in applying it to actual tea-picking scenarios.

On the one hand, most of the above papers are based on the picking scene of tea gardens with flat terrain. Therefore, the growth environment of tea trees is similar and the characteristics of tea buds are uniform, resulting in poor detection of tea buds in complex environments. Unlike the dataset production mentioned above, the dataset production scenario in this article is created from mountainous areas. Due to the influence of lighting and obstruction, there are significant differences in the growth of tea trees. Therefore, the model trained on the dataset in this article is more capable of handling complex scenarios. On the other hand, in order to improve the *precision*, *recall*, and mean average *precision* of the model, this paper optimizes the focus blur of the shooting equipment and addresses the problem of different tea bud sizes. At the same time, it can ensure that the model can be easily deployed to picking equipment. The specific contributions are as follows:

(1) The dataset was collected in mountainous areas, and data were collected on spring tea under different growth conditions on sunny and rainy days. Data for one bud, one bud with one leaf, and one bud with two leaves were annotated based on the size of the tender buds.

(2) SimSPPF is introduced into the backbone network, replacing serial connections with parallel connections based on SPP. Additionally, the activation function is replaced with a simpler ReLU to improve the computational efficiency of the model and facilitate deployment on picking equipment.

(3) To solve the problem of blurred focus in shooting equipment, a BiFPN bidirectional connection mechanism is used in the neck network to enhance the network’s feature expression and improve the model’s accuracy in identifying tea buds with blurred focus.

(4) Due to the terrain factors of tea tree planting, some tea bud targets are too small. Replacing traditional convolution modules with ODConv in the neck allows the model to focus on more dimensions of information, thereby improving the accuracy of small target tea bud detection.

## 2. Materials and Methods

### 2.1. Data Acquisition

**Figure 1:**
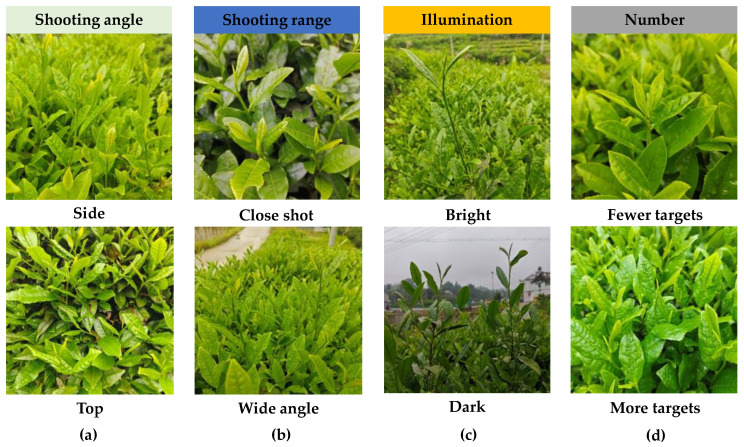
Tea sprout images under different conditions: (**a**) shooting angle: side and top; (**b**) shooting range: close shot and wide angle; (**c**) illumination: bright and dark; (**d**) number: fewer targets and more targets.

The dataset of tea buds used in this research was collected in the West Tea Valley of Youfangdian, Jinzhai County, Lu’an City, Anhui Province, China, and the object of the collection was Lu’an Gua Pian tea in spring. The resolution of the data is 3072 × 3072 pixels, saved in JPG format. The data collection time is early April 2024, and the shooting time is from 08:00 to 18:00. When collecting images of tea buds, the distance between the shooting equipment and the tea tree is 10–50 cm, and the shooting angle is kept within a certain range relative to the ground angle. The perspective of the tea-picking machine is imitated as much as possible for scene shooting. A total of 4100 tea bud targets were collected, as shown in Figure 1, which includes datasets with different shooting angles, near and far views, different lighting intensities, different angles, and different numbers of targets in a single image.

### 2.2. Version Selection of YOLOv5

Yolov5 is a single-stage object detection model developed based on the YOLO (You Only Look Once) series [18]. The backbone of YOLOv5 uses the CSPDarknet53 network, which is relatively lightweight and can reduce the computational load during training while ensuring high detection accuracy. It is mainly used to convert input images into multi-layer feature maps. The neck section adopts a combination of FPN and PAN pyramid structures to handle targets of different scales and sizes. The detection head is composed of multiple convolutional layers, responsible for predicting bounding boxes, target scores, and category probabilities. YOLOv5 directly predicts bounding boxes without the need for pre-anchor boxes, greatly simplifying the training process and improving the model’s ability to generalize to objects of different shapes and sizes [19].

According to the number of residual structures, model depth multiple, and layer channel multiple, YOLOv5 can be divided into four versions: YOLOv5s, YOLOv5m, YOLOv5l, and YOLOv5x. The dataset created in this article is used for the above four versions to test the performance of different versions and facilitate the selection of version parameters suitable for the research content. The test results are shown in Table 1. Among them, YOLOv5s has the smallest parameters and FLOPs, and *mAP* is not significantly different from other versions. Although YOLOv5x and YOLOv5l have the highest *mAP*, their parameters and FLOPs far exceed other versions, which is not conducive to deployment on tea-picking equipment and requires lightweight processing [20]. The parameters of YOLOv5m are three times those of YOLOv5s, but *mAP* only increases by 1.7%. To balance the size and accuracy of the model, this study chose YOLOv5s with a model depth multiple of 0.33 and layer channel multiple of 0.5 as the base model. While being able to quickly reason and ensure accuracy, it is convenient for deployment on mobile devices. The improvement in subsequent models will also be based on YOLOv5s.

### 2.3. Improved YOLOv5-Tea Model

**Figure 2:**
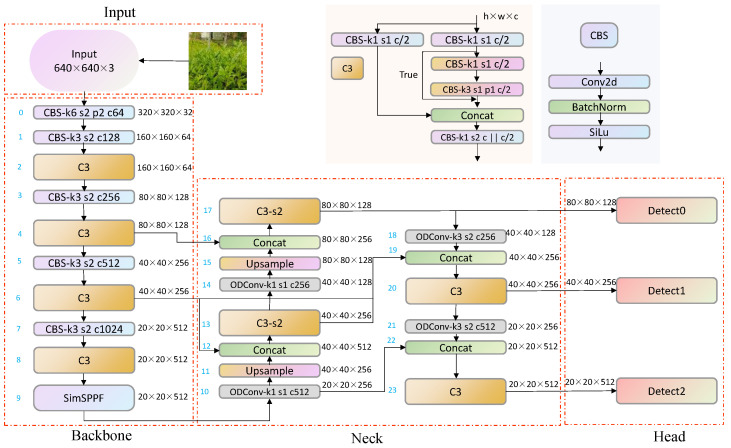
Improved YOLOv5-tea network structure.

To ensure minimal changes in the improved model parameters and FLOPs while improving the model’s accuracy metrics, this article proposes a model to further improve the network’s feature extraction and feature fusion abilities. It also improves the detection performance of the model for fuzzy tea buds and small target tea buds. Figure 2 shows the network structure of the improved YOLOv5-tea. The network receives input images, scales them to 640 × 640, and normalizes them. It uses a CSPNet (Cross-Stage Partial Network) as the backbone, dividing the feature map into multiple parts and merging them at different stages to improve the efficiency and performance of feature extraction. Further processing of features in the neck enhances information at different scales, using BiFPN for better fusion of features at different scales. Finally, the head section is used to generate the coordinates and confidence of the bounding box.

Compared to the SPP module in YOLOv4, the SimSPPF in this network switches from parallel to series to reduce model complexity. By reducing parameters and computational complexity to a small extent, there is a relatively fast feature extraction speed. Compared to the SPPFCSPC module in YOLOv7, although SPPFCSPC has better detection performance for object detection, the complexity of the model has increased dramatically, and the number of network parameters has increased by 47%. This does not conform to this article’s original intention of deploying the model on mobile devices. Comparing the FPN structures of YOLOv4 and YOLOv7, in this paper, BiFPN simplifies the network nodes, preserves the information of low-resolution feature maps, flexibly integrates features of different scales, and solves the problem of tea bud recognition with unclear focus. The convolution modules of YOLOv4 and YOLOv7 are both based on a convolutional layer, a batch normalization layer, and an activation function, which statically extract features. ODConv not only involves multiple dimensions but also dynamically adjusts the number of convolution kernels. This convolution is efficient while ensuring complementarity and synergy between dimensions.

#### 2.3.1. Simplified Spatial Pyramid Pooling Fast

**Figure 3:**
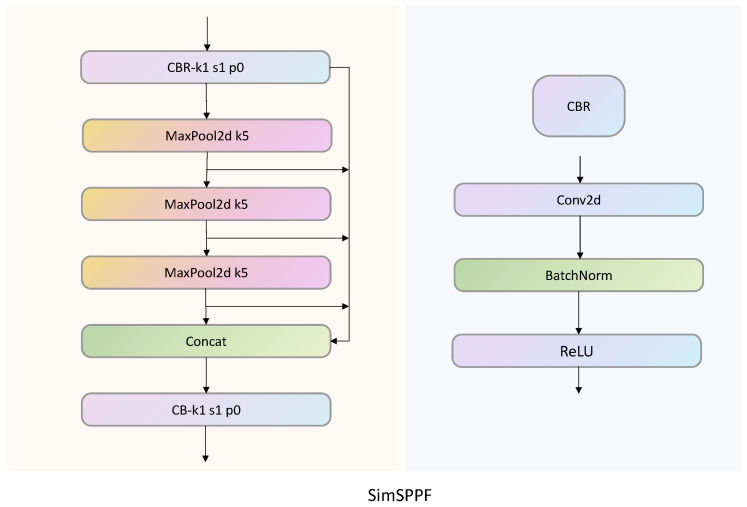
SimSPPF module structure diagram.

In the study by He et al. [21], the Spatial Pyramid Pooling (SPP) module is proposed. In the module, three max-pooling kernels are connected in parallel to achieve the fusion of local and global features. This method can avoid the repeated calculation of convolutional features, improve the speed of generating candidate boxes, and save computational costs. On the basis of the SPP module, YOLOv5 proposes SPPF, with the main difference being that the pooling kernels in SPP are connected in series instead of parallel. This method greatly reduces the computational load of the model through multi-layer pooling and can also improve the model’s speed. The main difference between the SimSPPF used in this article and the SPPF module is the activation function used inside the module. As shown in Figure 3, the activation function used by SPPF is the SiLU activation function, while SimSPPF uses the ReLU activation function. Due to the use of only 2D max pooling with a pooling kernel of 5, compared to traditional modules with pooling kernels of 5, 9, and 13, max pooling can reduce computational complexity. At the same time, by using a parallel residual structure, the pooled features are extracted into Concat and continue with the next step of pooling to obtain features at different levels, thereby improving the model’s ability to extract input image features.

To verify the better advantages of introducing SimSPPF for tea bud detection, this study conducted comparative experiments between the YOLOv5 and YOLOv5 SimSPPF networks with the SimSPPF module added. As shown in Table 2, after replacing the SimSPPF module, both the FPS (frames per second) and the accuracy of the model were improved, while the model parameters remained basically unchanged.

#### 2.3.2. Bidirectional Feature Pyramid Network

**Figure 4:**
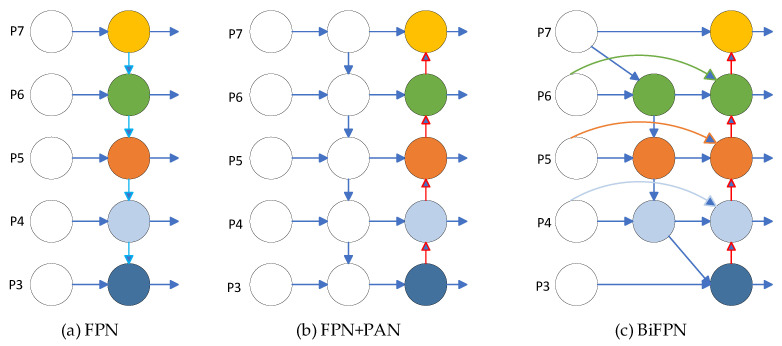
(**a**) FPN network architecture; (**b**) FPN + PAN network structure; (**c**) BiFPN network architecture.

In the process of network training, due to the different sizes of tea buds, there are more pixels for large targets and fewer pixels for small targets. As the convolutional layer deepens, the features of small targets will gradually disappear, and only the features of large targets will be retained. In order to integrate features from different layers and improve the impact of scale issues, the YOLOv5 algorithm uses the FPN + PAN structure as the feature fusion network. The function of FPN is to build a top–down channel, which will lose positional information while ensuring high semantic information in the upper-level feature map [22], as shown in Figure 4a. On the basis of FPN, a bottom–up path PAN structure is added to enhance the network’s location and semantic information and improve its object detection accuracy [23], as shown in Figure 4b. The disadvantage of this cascading method is that it requires feature maps to be of the same size, and information of different scales cannot be learned, which limits the improvement in detection accuracy. To address this issue, this paper introduces a more efficient BiFPN into the network to further improve the accuracy of object detection [24]. The specific structure is shown in Figure 4c.

BiFPN has the following improvements compared to FPN + PAN: In terms of cross-scale connectivity, BiFPN removes nodes with only one input and establishes a new path between input nodes and output nodes at the same level. To achieve better feature fusion, BiFPN is repeatedly stacked as the base layer. Cross-scale connections not only fuse more features without increasing computational complexity but also improve accuracy with minimal impact on model load. In terms of weighted feature fusion, FPN + PAN networks convert images of different resolutions to the same resolution and then concatenate them when fusing features of images of different resolutions. But different resolutions carry different input features, and unifying the resolution will inevitably result in the loss of features from the unified image. In order to avoid the loss of feature information, the BiFPN network multiplies the input features with different weights and removes the softmax operation to improve the speed of model training.

Using the BiFPN structure as the neck network for feature fusion can solve the following problems: One is the problem of detecting small tea buds, which helps to detect shallow features of tea buds. The second is the problem of tea buds being obscured. By using cross-scale connections, different features can be suppressed or enhanced according to cross-scale weights to reduce the problem of tea buds being blurry and unrecognizable.

#### 2.3.3. Omni-Dimensional Dynamic Convolution

In the field of deep learning, in order to improve the performance of network models, methods such as expanding the network width, depth, resolution, etc., are generally used to increase the model size. Moreover, the convolution kernels used in convolution are independent of the input dataset samples, which undoubtedly poses a great obstacle to the convergence performance of the network. In the study by Yang et al. [25], the authors first proposed dynamic convolution. Different from the weighting method applied to feature maps, CondConv linearly weights multiple convolution kernels to make the weights related to the input and improve the dependence of dynamic convolution on input data. In short, it involves using different convolution kernels for different inputs and then weighting the attention of these different kernels.

**Figure 5:**
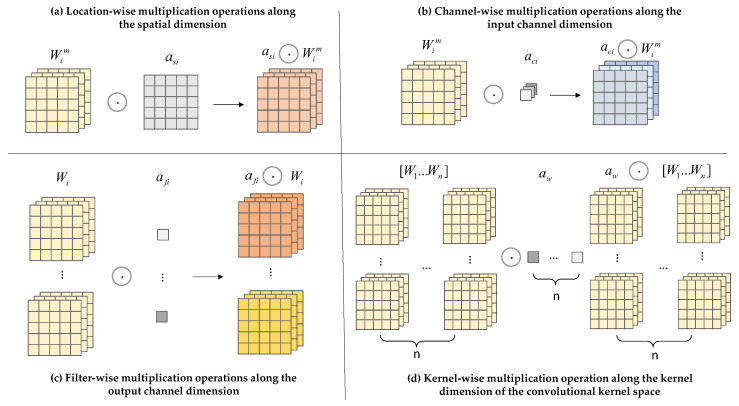
Illustration of the operations of the four different sorts of weights used in ODConv.

**Figure 6:**
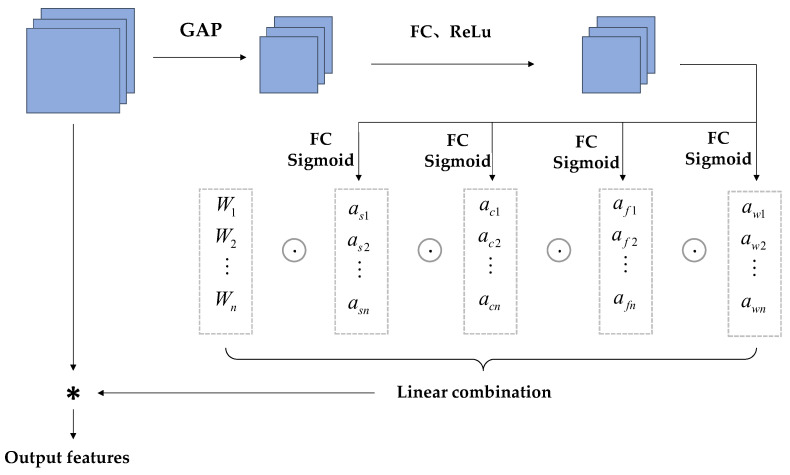
Structure of ODConv layer. Explation: “*” stands for multiplication.

CondConv convolution relies on using the number of convolution kernels to assign dynamic properties to the kernels. To avoid the aforementioned issues, ODConv is used in the model. It processes the network performance gain using four different weights: spatial size, input channel, output channel, and convolution kernel [26], as shown in Figure 5. The specific architecture is shown in Figure 6. The input features are first processed in the Global Average Pooling (GAP) layer. The input is generated after being fed into the fully connected (FC) layer and the ReLu activation function layer, and then fed into the four different weights shown in the figure below for processing. After this series of processing, ODConv can improve the diversity of tea bud feature extraction and greatly enhance the network feature extraction capability. The reason for choosing the GAP layer to process input features is that the GPA layer does not have parameters for optimization, which can reduce or avoid overfitting. Although hindered in capturing spatial capabilities, average pooling sums up spatial information and is more robust to input spatial variations. It can be applied to complex tea bud-picking scenarios.

Among them, as shown in Figure 5a, $a_{si}$ is the attention along the dimension of the convolutional kernel space, and different attention values are assigned to convolutions of k × k. As shown in Figure 5b, $a_{ci}$ represents the attention along different channel dimensions of the input. As shown in Figure 5c, $a_{fi}$ represents the attention along different channel dimensions of the output. As shown in Figure 5d, $a_{wi}$ is the attention that focuses on n convolutional kernel dimensions. The four different dimensions are sequentially multiplied by the convolution kernel $W_{i}$ and accumulated, complementing each other to improve the model’s ability to capture details. The definition of dynamic convolution in ODConv is shown in Formula (1).

(1) $$y=(a_{w1}⊙a_{f1}⊙a_{c1}⊙a_{s1}⊙W_{1}+\ldots +a_{\mathrm{wn}}⊙a_{fn}⊙a_{cn}⊙a_{sn}⊙W_{n})\times x$$

For tea bud detection, the CSB convolution module focuses more on shallow edge feature information, which is also a key factor in distinguishing tea buds [27]. ODConv focuses more on deeper features such as tea bud texture and color. Therefore, a combination of feature extraction from two modules should be taken in the model. This article chooses to use traditional CSB convolution modules for shallow feature extraction in the backbone section and ODConv convolution in the neck section. Using the BiFPN structure mentioned earlier for feature fusion, which takes into account features at different stages, has a significant effect on improving the accuracy of tea bud detection. As shown in Table 3, YOLOv5-all indicates that all independent CSB convolution modules in the YOLOv5 model have been replaced with ODConv. YOLOv5-ODConv only replaces the independent CSB module of the neck. Comparing the two sets of data, it can be found that replacing all CSB modules in the network with ODConv will ignore too much information from the shallow network, resulting in a decrease in detection accuracy. Therefore, when improving the original network, this article chooses to improve it using YOLOv5-ODConv.

### 2.4. Experimental Procedures

#### 2.4.1. Experimental Environment

All experiments in this study were based on the PyTorch deep learning framework and programmed in Python. The software, hardware environment, and parameters used in the experiments are shown in Table 4.

#### 2.4.2. Experimental Details

YOLOv5 is used as the initial program, and the above method is used to improve the program. The training process uses the stochastic gradient descent (SGD) optimizer, with an initial learning rate set to 0.01, a final learning rate set to 0.00001, and an SGD momentum set to 0.937. Using the cosine annealing scheduler to gradually reduce the learning rate helps the model converge stably and improves training effectiveness. The formula is shown in (2), where $lr_{new}$ is the new learning rate, $lr_{initial}$ is the initial learning rate, $epoch_{cur}$ is the value of the current trained epoch, and $epoch_{t}$ is the total number of trained epochs. Divide the training set and the validation set in an 80%/20% ratio, scale the input image pixels to 640 × 640 as specified by the network, set the epoch to 300, and set the batch size to 32.

(2) $$lr_{new}=lr_{initial}\times ((1+\cos(\frac{epoch_{cur}}{epoch_{t}}\times \pi ))/2)$$

The experimental networks were enhanced with mosaic, HSV, and fliplr data. Mosaic data augmentation enriches the dataset by randomly scaling, cropping, and stitching four images; HSV data augmentation transforms images in terms of Hue (H), Saturation (S), and Value (V); and fliplr flips images left and right. The purpose is to expand the dataset and improve the generalization ability of the object detection model. In order to ensure the accuracy of each comparative experiment, YOLOv5-tea and the comparative network did not load pre-trained weights. After mechanical picking, unidentified tea buds need to be manually picked in the tea garden. A higher *recall* can reduce the cost of manual picking, so this article sets the *recall* and *precision* ratio to 3:2 when selecting the weight file.

#### 2.4.3. Evaluation Indicators

To effectively evaluate the model performance, the model was evaluated using *precision*, *recall*, and Mean Average Precision (mAP) as evaluation indexes for model recognition of tea buds.

(3) $$Precision=\frac{\mathrm{TP}}{\mathrm{TP}+\mathrm{FP}}$$

(4) $$Recall=\frac{\mathrm{TP}}{\mathrm{TP}+\mathrm{FN}}$$

(5) $$mAP=\frac{\sum_{1}^{N}AP}{N}=\frac{\sum_{1}^{N}\int_{0}^{1}P(R)dR}{N}$$

where TP is true positive, FP is false positive, and FN is false negative. *Precision* is the probability that a tea bud is correctly predicted as a positive sample in a sample predicted as a positive sample; *recall* is the probability that a tea bud is finally correctly predicted as a positive sample in a positive sample of the original sample. AP is the average *precision*, denoted denoted by Precision and Recall. mAP is the average *precision* mean, which is the average of the detected categories’ AP values. Since the detected objects consist only of tea buds, *N* is set to 1. The value of AP is equal to the value of mAP. The metrics used in this study are all mAP@50 (IoU threshold is 0.5). When there is at least 50% overlap between the detection box and the actual bounding box, the sample is positive.

## 3. Results and Discussion

### 3.1. Comparison Experiment Before and After Model Change

On the dataset constructed in this article, the original model and the YOLOv5-tea model were compared, and the experimental results are shown in Table 5. After the improvement, model P increased by 4.4%, R increased by 2.3%, *mAP* increased by 3.2%, floating-point operations (FLOPs) decreased by 1 G, and model parameters only increased by 0.23 M. From the data perspective, the improved 14.9 M model can be easily deployed on mobile devices, and after calculation, the 33.9 FPS can still meet real-time detection requirements.

**Figure 7:**
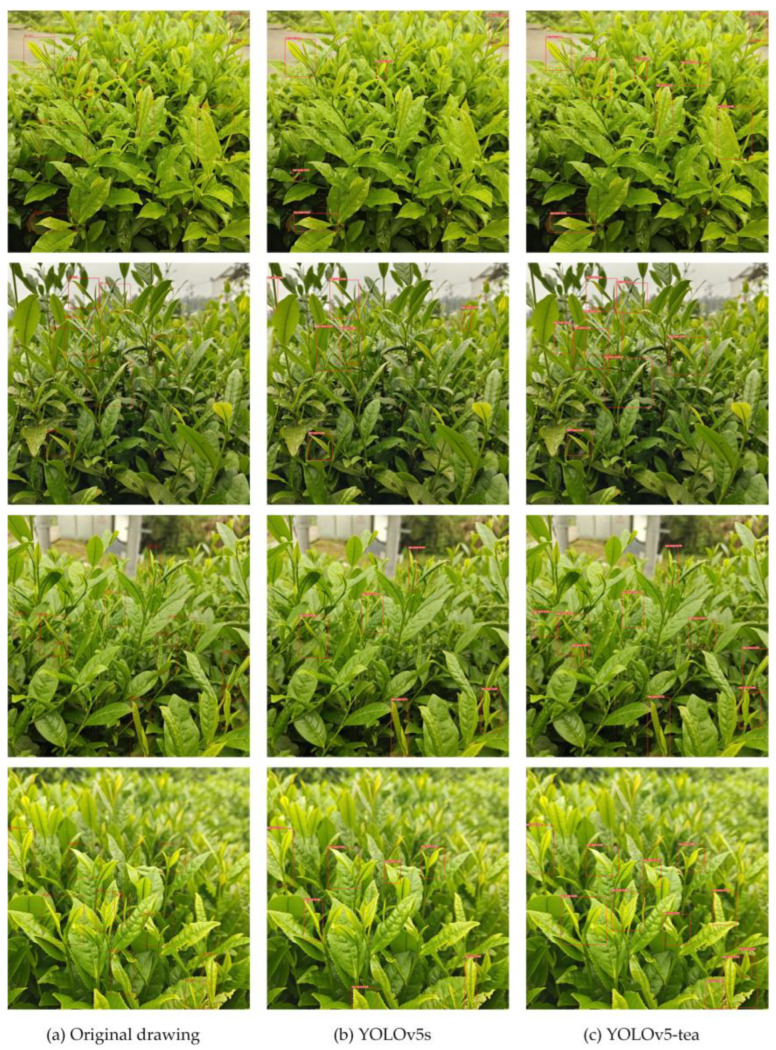
Result validation: The red box represents the tea buds detected by the model. (**a**) original drawing; (**b**) detected by YOLOv5s model; (**c**) detected by YOLOv5-tea model.

Compared with the original model, the YOLOv5-tea model has improved its performance in detecting small target tea buds and occluded tea buds. Figure 7 shows four randomly selected tea bud images. From the comparison of detection boxes (b) and (c), it can be seen that YOLOv5-tea has a certain improvement in the detection performance of small targets and focused blurry targets.

### 3.2. Model Ablation Studies

To verify the effectiveness of the improved YOLOv5-tea network model for tea bud target detection, ablation comparisons were conducted on each module, and the results are shown in Table 6. From the table, it can be seen that, compared with the YOLOv5 model, replacing SimSPPF, BiFPN, and ODConv separately on the basis of the original model significantly improves the *mAP* of the model. Replacing with SimSPPF not only increased *mAP* by 1.3% but also slightly improved FPS. Replacing the network connection structure of the neck part with BiFPN, due to the addition of cross-level feature learning in the BiFPN structure, resulted in an increase in parameters and FLOPs by 0.15 M and 0.6 G, respectively, but the evaluation metrics improved. After replacing the traditional convolutional structure in the neck with ODConv, FLOPs decreased by 1.1 G and *mAP* increased by 1.9% compared to the original model. After combining different improvements in different ways, the evaluation indicators reached the highest level. It can be seen that, compared to the original model, this paper’s YOLOv5-tea model improved evaluation indicators P, R, and *mAP* by 4.4%, 2.3%, and 3.2%, respectively, while FLOPs decreased. From the results of the ablation comparison experiment, it can be seen that all the improvements made in this study based on YOLOv5s played their due role compared to the original model.

### 3.3. Experimental Comparison of Different Detection Algorithm Models

**Figure 8:**
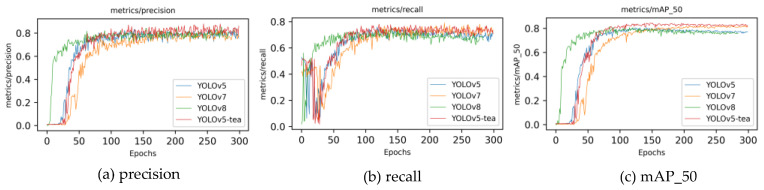
Metric evolution during model training across various models: (**a**) *precision*; (**b**) *recall*; (**c**) *mAP*_50.

To validate the superiority of the YOLOv5-tea model, an improvement over YOLOv5, this study compares its performance metrics with other leading algorithm models. Given that the two-stage algorithm network framework model is too large to deploy to lightweight devices and cannot achieve real-time detection, this article mainly compares the single-stage YOLO algorithm series. The model depth multiple is set to 0.33, the layer channel multiple is set to 0.5, and the learning rate and YOLOv5-tea are kept consistent. As shown in Table 7, the performance indicators of YOLOv5, YOLOv6, YOLOv7, and YOLOv8 are compared. From the results, YOLOv5-tea has the highest detection accuracy. Figure 8 shows the changes in various indicators during the model training process. YOLOv5-tea, represented by the red line, has a significantly higher *mAP* than other models after convergence, and its convergence speed is also in the middle position. Through the modifications in this article, multiple indicators of the model have been improved.

## 4. Conclusions

For machine equipment, the tea bud recognition model not only needs to consider the inference speed of the model but also the accuracy of the model, which is very important. In the future, when the model needs to be deployed to mobile devices, there will inevitably be disadvantages such as low hardware computing power and poor performance. Therefore, it is necessary to balance the accuracy and speed of tea bud detection. To adapt to real-world tea-picking scenarios, this study conducts a series of optimizations on YOLOv5. The *precision* of the tea bud model is 84.5%, the *recall* is 74.1%, *mAP* is 83.7%, and the model size is only 14.9 MB, making it convenient to deploy detection models on mobile devices with limited computing power and storage space. Compared with other mainstream models, the improved model in this paper has the advantages of less computation, smaller model size, and higher detection accuracy. In this study, the dataset collected tea bud images from various environments, including different lighting conditions and angles, which made the model robust. This article proposes a new optimization direction for the tea bud detection algorithm and achieves good performance in an experimental structure, providing certain assistance for the development of intelligent tea picking. The follow-up work will be based on optimizing the picking model of premium tea and continuously improving the detection speed and accuracy.

In future research, we will rely on the Anhui Province Forest Crop Intelligent Equipment Engineering Platform to collect tea bud data for different tea trees and seasons, enriching the depth of the research topic. At the same time, we will pay attention to more evaluation indicators to avoid imbalanced datasets. Firstly, we will determine the picking time for green tea, white tea, and black tea throughout the year and collect data on the corresponding tea leaves during the corresponding time periods. Based on multiple evaluation indicators such as *P*, *R*, *mAP*, and F1, the optimal model will be determined comprehensively, while ensuring that the model is lightweight enough to achieve higher generalization performance. Finally, based on the use of our platform in real tea-picking scenarios, the model will be dynamically adjusted according to the situation.

## Figures and Tables

**Table 1:** Performance and parameters of different models of YOLOv5.

Model	Depth Multiple	Width Multiple	Parameters (M)	FLOPs (G)	*mAP* (%)
YOLOv5s	0.33	0.50	7.01	15.8	80.5
YOLOv5m	0.67	0.75	20.85	47.9	82.2
YOLOv5l	1.0	1.0	46.11	107.6	84.2
YOLOv5x	1.33	1.33	86.17	203.8	84.9

**Table 2:** Comparison experiment between YOLOv5 and YOLOv5-SimSPPF.

Model	Parameters (M)	FLOPS (G)	Speed (FPS)	Size (M)	*mAP* (%)
YOLOv5	7.01	15.8	42.92	14.5	80.5
YOLOv5-SimSPPF	7.01	15.8	43.86	14.5	81.8

**Table 3:** Comparison experiment between YOLOv5-all and YOLOv5-ODConv.

Model	Parameters (M)	FLOPs (G)	Size (M)	Speed (FPS)	*mAP* (%)
YOLOv5-all	7.12	11.0	14.7	31.65	81.7
YOLOv5-ODConv	7.09	14.7	14.6	33.90	82.4

**Table 4:** Software and hardware parameters.

Configuration	Parameter
CPU	AMD EPYC 7H12 64-Core Processor
GPU	NVIDIA GeForce RTX 3090
Operation system	Windows 10
RAM	512 GB
Development environments	Python3.8, Pytorch1.9.0, CUDA11.1

**Table 5:** Comparison experiment between YOLOv5 and YOLOv5-tea.

Model	Parameters (M)	FLOPs (G)	Size (M)	*P* (%)	*R* (%)	*mAP* (%)
YOLOv5	7.01	15.8	14.5	80.1	71.8	80.5
YOLOv5-tea	7.24	14.8	14.9	84.5	74.1	83.7

**Table 6:** Ablation comparative experiment on data annotation.

Model	Parameters (M)	FLOPs (G)	*P* (%)	*R* (%)	*mAP* (%)
YOLOv5	7.01	15.8	80.1	71.8	80.5
YOLOv5_SimSPPF	7.01	15.8	80.6	74.4	81.8
YOLOv5_BiFPN	7.16	16.4	81.4	73.4	82.4
YOLOv5_ODConv	7.09	14.7	83.0	71.3	82.4
YOLOv5_SimSPPF_BiFPN	7.16	16.4	84.1	72.6	82.6
YOLOv5_SimSPPF_ODConv	7.09	14.7	83.1	72.8	82.6
YOLOv5_BiFPN_ODConv	7.24	14.8	84.1	73.9	82.7
YOLOv5-tea	7.24	14.8	84.5	74.1	83.7

**Table 7:** Experimental comparison of different detection algorithm models.

Model	Parameters (M)	FLOPs (G)	Size (M)	*mAP* (%)
YOLOv5	7.01	15.8	14.5	80.5
YOLOv7	9.32	26.7	19.0	81.1
YOLOv8	11.13	28.4	22.5	81.6
YOLOv5-tea	7.24	14.8	14.9	83.7

## Data Availability

The raw data supporting the conclusions of this article will be made available by the authors upon request.
